# *Lichtheimia ramosa*: A Fatal Case of Mucormycosis

**DOI:** 10.1155/2016/2178218

**Published:** 2016-03-29

**Authors:** Cecilia Mouronte-Roibás, Virginia Leiro-Fernández, Maribel Botana-Rial, Cristina Ramos-Hernández, Guillermo Lago-Preciado, Concepción Fiaño-Valverde, Alberto Fernández-Villar

**Affiliations:** ^1^Pulmonary Department, Hospital Álvaro Cunqueiro, EOXI Vigo, NeumoVigoI+i Research Group, Vigo Biomedical Research Institute (IBIV), Estrada Clara Campoamor No. 341, 36312 Vigo, Spain; ^2^Critical Care Department, Hospital Álvaro Cunqueiro, EOXI Vigo, Estrada Clara Campoamor No. 341, 36312 Vigo, Spain; ^3^Pathology Department, Hospital Álvaro Cunqueiro, EOXI Vigo, Estrada Clara Campoamor No. 341, 36312 Vigo, Spain

## Abstract

Mucormycosis due to* Lichtheimia ramosa* is an infrequent opportunistic infection that can potentially be angioinvasive when affecting inmunocompromised hosts. We present a fatal case of mucormycosis, affecting a 56-year-old male with diabetes mellitus and siderosis, initially admitted to our hospital due to an H1N1 infection. The subject's clinical condition worsened and he finally died because of a necrotizing bilateral pneumonia with disseminated mycotic thromboses due to* Lichtheimia ramosa*, which is an emerging Mucoralean fungus. This is an infrequent case because of the extent to which it affected a subject without overt immunocompromise. This case underlines the importance of an early premortem diagnosis and treatment in order to prevent rapid progression of this disease, as well as the need of considering mucormycosis when facing subjects with multiple emboli and fever unresponsive to usual antimicrobials.

## 1. Case Presentation

A 56-year-old man was admitted with a one-week history of nonproductive cough, myalgias, dyspnoea, and fever (39°C). The subject was a former smoker, with a daily intake of 104 g of alcohol. His past medical history included well-controlled type 2 diabetes mellitus and siderosis, which had been diagnosed by bronchoscopy after finding a mild interstitial pattern in the CT. Lung function was normal at all times and the patient was never treated with oral corticosteroids before hospital admission. He also suffered from left renal agenesis. A chest radiograph showed a bilateral pneumonia and empirical treatment with meropenem and levofloxacin was started. Oseltamivir was also started empirically, given that this subject was admitted during later winter and at high risk of suffering an infection caused by the H1N1 influenza virus.

**Figure 1:**
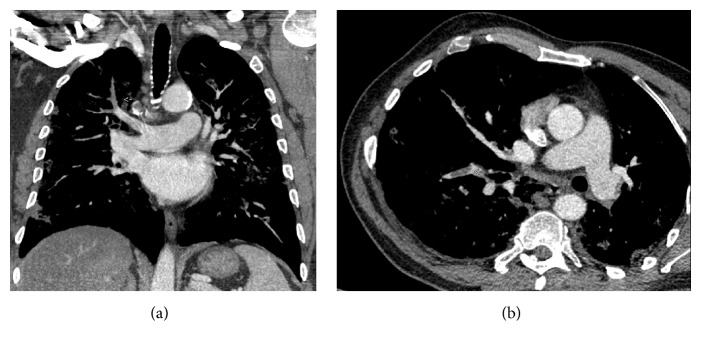
Pulmonary thromboembolism (arrow).

Worsening of the subject's clinical condition prompted the performance of a computed tomography (CT) 5 days after admission, which confirmed the diagnosis of pneumonia and incidentally found an acute pulmonary bilateral thromboembolism (Figures 1(a) and 1(b)). Low molecular weight heparin was started and oseltamivir was suspended after a 5-day treatment due to negative H1N1 nasal swabs.

A bronchoscopy was performed on the 7th day after admission and due to positive sputum cultures for* Streptococcus pyogenes*, the antibiotic therapy was switched to meropenem and cotrimoxazole. Transbronchial biopsies showed nonspecific acute alveolar damage.

**Figure 2:**
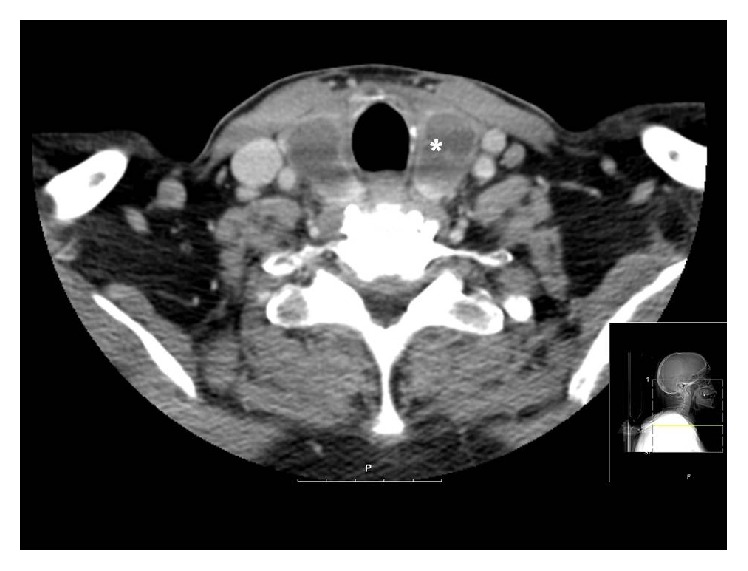
Subacute thyroiditis (*∗*).

After 9 days of progressive recovery, the subject's clinical condition worsened again, with fever, high acute phase reactants, leukocytosis, odynophagia, and persistent neck pain. Antibiotics were switched again to meropenem and linezolid and a neck-CT plus a neck ultrasonography were performed, with findings suggestive of a de Quervain subacute thyroiditis (Figure 2). High-dose corticosteroids were started (1.5 mg/kg/24 h).

**Figure 3:**
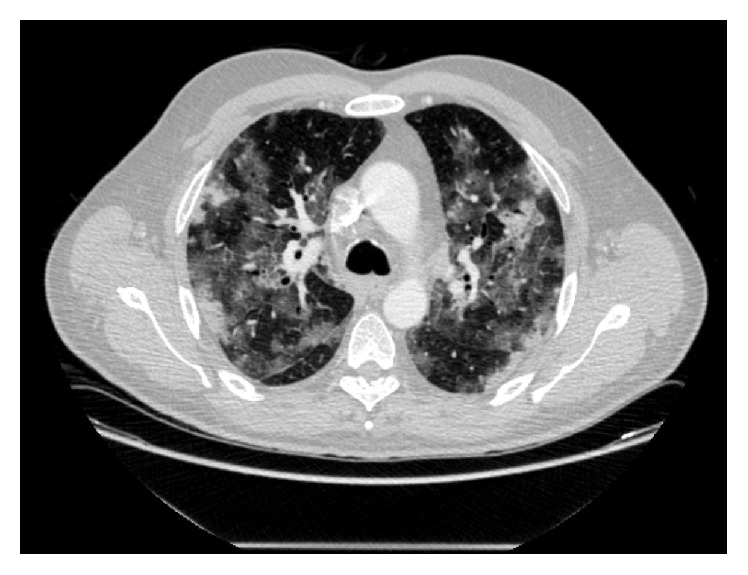
Pulmonary parenchymal damage.

Another thoracic CT was performed 18 days after admission, showing multiple cavitary nodules and masses in both lungs (Figure 3), as well as hypodense areas in the right kidney and a diffuse hypodensity in the thyroid. Acute renal failure developed the same day with adjustments to fluid therapy subsequently made.

Voriconazole was empirically started for a presumed fungal infection, and both transthoracic and transesophageal echocardiograms were negative for endocarditis. Serologic tests for HIV and B and C hepatitis were negative, as were autoimmune markers such as ANA and ANCA. A bone marrow biopsy was also performed but did not show any sign of hematologic malignancy.

**Figure 4:**
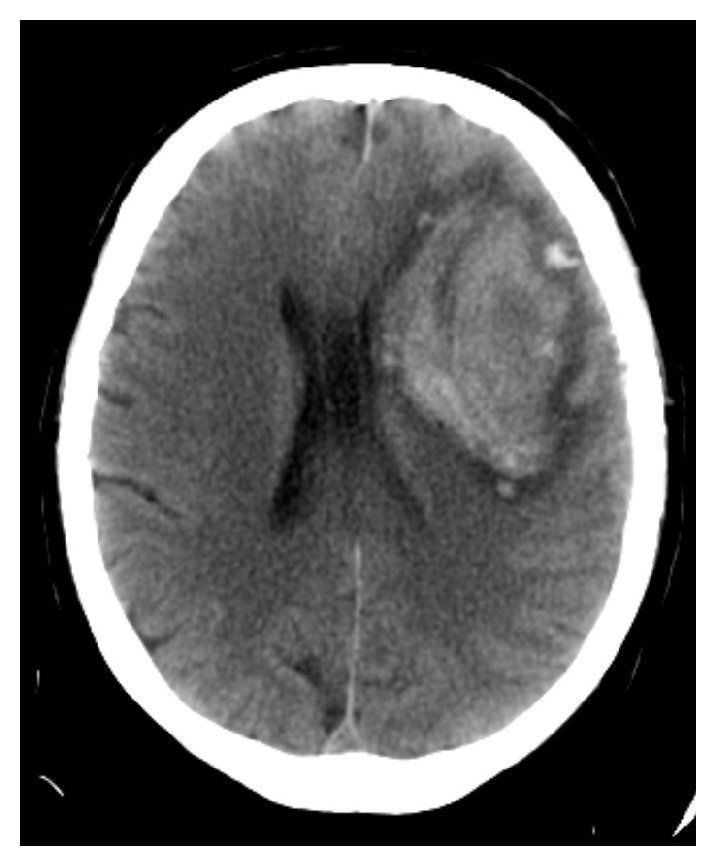
Left frontal hematoma.

On the third day of established kidney dysfunction, haemodialysis was started. On the 25th day after admission, the subject's respiratory condition worsened again, requiring invasive ventilation. On the 26th day after admission, the subject suffered from neurologic worsening, consisting of a decline of consciousness, with a GCS of 7 and a left unreactive pupil. A cranial CT showed a left frontal hematoma with mass effect (Figure 4), prompting urgent neurosurgery. After the intervention, there were signs of haemorrhagic shock despite the use of fluid therapy, blood products, norepinephrine, and dopamine, with progressive abdominal distension. An urgent abdominal CT was performed, with active bleeding through distal pancreatic-duodenal vessels that was refractory to embolization. The subject died after developing refractory shock.

Necropsy results demonstrated invasive mucormycosis due to* Lichtheimia ramosa*, with mycotic thrombotic embolisms in the lungs (with a necrotizing bilateral pneumonia producing secondary systemic invasion through mycotic thrombosis), kidney, brain, thyroid, heart, liver, spleen, mediastinal lymph nodes, bladder, ureter, and small bowel.

## 2. Discussion

Mucormycosis is an opportunistic invasive infection caused by fungi of the order Mucorales, like* Mucor*,* Rhizopus, or Lichtheimia* (previously known as* Absidia*). It manifests in a variety of syndromes, affecting mainly immunocompromised subjects (especially those with hematologic malignancies) and those with diabetes mellitus, iron overload, and under treatment with glucocorticoids and it can also be manifested by cutaneous and subcutaneous disease in some trauma patients without known immunocompromise. These fungi are ubiquitous, and both rhino-orbital-cerebral and pulmonary mucormycosis are acquired by the inhalation of their spores. They are potentially angioinvasive; thus the disseminated disease is characterized by vascular invasion and thrombosis. Despite their low virulence,* Lichtheimia* species are currently regarded as emerging pathogens among Mucoralean fungi [1].

We are presenting a fatal case of invasive mucormycosis in a subject where the main underlying condition was diabetes mellitus, although it was well-controlled, and without traces of ketoacidosis. Some authors [2] have presented cases demonstrating that mucormycosis can occur in healthy individuals without any contributory factors; therefore even well-controlled diabetes may represent a risk factor for Mucoralean infections. This review [3] has found a significant association between haematological malignancy and lung involvement or disseminated infection and between diabetes mellitus and rhinocerebral infection. The association between diabetes mellitus and lung or disseminated infection is less likely, although we have to keep in mind that our subject had been taking high-dose glucocorticoids during hospital stay and also had an underlying lung disease. We have found no references about the association between siderosis as an underlying condition and the development of invasive fungal infections.

It is possible that our subject had a healthcare associated mucormycosis after having been admitted at our hospital for an H1N1 infection with a secondary pneumonia, although we did not search for any particular portal of entry and, in any case, Mucoralean fungi are ubiquitous. In this review [4], 22% of the subjects with healthcare associated mucormycosis had diabetes mellitus as an underlying risk factor, and only 8% of infections were due to* Lichtheimia*, although this species of Mucoral fungi should be regarded as an emerging pathogen according to recent reviews [1]. Nevertheless, it should be considered that there are regional differences in the epidemiology which suggest that this fungus might be higher on the differential for European patients than North American patients.

This case report shows that seemingly inmunocompetent individuals can develop fatal fungal infections. The necropsy showed thrombotic mycosis in the lungs, kidney, brain, thyroid, heart, spleen, liver, lymph nodes, bladder, ureter, and small bowel. The two typical presentations are rhino-orbital-cerebral and pulmonary disease [5], and once they disseminate, the most frequent locations affected are those near to the origin. This case is an example of how difficult it is to make an early antemortem diagnosis, thus leading to rapid systemic dissemination and death.

In subjects who develop features suggestive of widespread thrombosis and infarction and a fever unresponsive to antibacterial antibiotics, fungal infections (including mucormycosis) should be considered in order to make an early diagnosis [6]. In addition, it should be noted that the clinical course and the imaging of invasive* Lichtheimia ramosa* are similar to those of an invasive aspergillosis. It is important to think in mucormycosis as well as in aspergillosis, as the antifungal agents appropriate for each infection are different [7].

Amphotericin B is the preferred empiric treatment for* Lichtheimia ramosa* and other mucormycosis.* Lichtheimia* is usually sensitive to posaconazole, which is possibly the preferred oral therapy. Amphotericin B is the most active drug against* Lichtheimia* species, with a geometric mean in vitro of 0.07 mg/liter at 24 h, while posaconazole had the highest activity among the azoles (geometric mean of 0.34 mg/liter at 24 h) [8]. Treatment of mucormycosis involves a combination of empirical antifungal treatment and surgical debridement of involved tissues [9].

In conclusion, the case we present represents an infrequent entity because of the causal species (*Lichtheimia ramosa*) and the fatality of the fungal infection in a seemingly inmunocompetent host. It summarises the difficulty of making an early diagnosis and the importance of considering mucormycosis when subjects develop widespread thrombosis and fever unresponsive to other antimicrobials.

## Learning Objectives

- Consider mucormycosis when multiple emboli and fever unresponsive to usual antimicrobial are present.
- Early premortem diagnosis in immunocompromised patients diminishes morbidity and mortality.

## Pretest

- When should a mucormycosis be suspected?
- How can we manage an infection due to* Lichtheimia ramosa*?

## Posttest

- When should a mucormycosis be suspected? Mucormycosis is an opportunistic infection which can be invasive when affecting immunocompromised hosts, causing vascular invasion and thromboses. Although some authors have presented cases demonstrating that mucormycosis can occur in healthy individuals without any contributory factors, even well-controlled diabetes may represent a risk factor for Mucoralean infections.
- How can we manage an infection due to* Lichtheimia ramosa*? The clinical course and the imaging of invasive* Lichtheimia ramosa* are similar to those of invasive aspergillosis. However, antifungal agents appropriate for each infection are different. Amphotericin B is the preferred empiric treatment for* Lichtheimia ramosa* and other mucormycoses.* Lichtheimia* is usually sensitive to posaconazole, which is possibly the preferred oral therapy. Treatment of mucormycosis involves a combination of empirical antifungal treatment and surgical debridement of involved tissues.
